# Supplementary material for: Effects of Green Tea Marinade in the Bioaccessibility of Tonalide and Benzophenone 3 in Cooked European Seabass
Source: Molecules. 2022 Jul 29;27(15):4873. doi: 10.3390/molecules27154873 (PMC9370058; doi:10.3390/molecules27154873)
Supplement: Supplementary file 1 [file molecules-27-04873-s001.zip › molecules-1805941-supplementary.pdf]

Effects of Green Tea Marinade in the Bioaccessibility of Tonalide and Benzophenone 3 in Cooked European Seabass

Sara C. Cunha [1,*], Juliana R. Gadelha [1], Flávia Mello [1], Isa Marmelo [2,3,4], António Marques [2,3] and José O. Fernandes [1]

[1] LAQV/REQUIMTE, Laboratório de Bromatologia e Hidrologia, Faculdade de Farmácia, Universidade do Porto, Rua de Jorge Viterbo Ferreira 228, 4050-313 Porto, Portugal; jgadelha@ua.pt (J.R.G.); flavia.bioufrj@gmail.com (F.M.); josefer@ff.up.pt (J.O.F.)
[2] IPMA, Portuguese Institute for the Sea and Atmosphere, I.P., Division of Aquaculture, Upgrading and Bioprospection (DivAV), Av. Doutor Alfredo Magalhães Ramalho 6, 1495-165 Lisboa, Portugal; isa.marmelo@ipma.pt (I.M.); amarques@ipma.pt (A.M.)
[3] CIIMAR, Interdisciplinary Centre of Marine and Environmental Research, University of Porto, Rua dos Bragas 289, 4050-123 Porto, Portugal
[4] UCIBIO-REQUIMTE, Applied Molecular Biosciences Unit, Department of Chemistry, NOVA School of Science and Technology, NOVA University of Lisbon, 2829-516 Caparica, Portugal
* Correspondence: sara.cunha@ff.up.pt; Tel.: +351-220428639; Fax: +351-226093390

Table S1- % Protein bioaccessibility in the sample analysed.

**Samples**	**% Protein bioaccessibility**
Roasted European seabass	91.1
Frying European seabass	98.3
Roasted European seabass previous marinade with green tea	90.3
Frying European seabass previous marinade with green tea	96.2

Table S2 - Sunflower vegetable oil composition (label information).

Fatty acids g/100 g	Saturated	10
	Monounsaturated	28
	Polyunsaturated	53
Tocopherols mg/100g		60

Table S3 - Catechin in green tea marinade (Henning et al., 2003).

**Tea Catechin**	**mg/100 mL**
Gallic acid	1.3 ± 0.61
Caffeine	29.0 ± 2.4 6
(-)-Epigallocatechin -EGC	80.2 ± 6.2
Catechin	4.9 ± 1.7
Epicatechin	16.3 ± 1.7
(-)-Epigallocatechin gallate- EGCG	83.3 ± 14.9
Gallocatechin gallate- GCG	3.3 ± 0.9
Epicatechin gallate- ECG	10.5 ± 3.2
Total catechins	201.4 ± 27.2

Henning, S., Fajardo-Lira, C., Lee, H., Youssefian, A., Go, V., & Heber, D. (2003). Catechin Content of 18 Teas and a Green Tea Extract Supplement Correlates with the Antioxidant Capacity. *Nutrition and Cancer*, *45*(2), 226-235. doi: 10.1207/s15327914nc4502_13.

Table S4 - Linearity range (coefficient of determination, recoveries (%), intraday and interday precision (%RSD), limits of detection (LOD) and quantification (LOQ) of the analytical method applied to determine tonalide (ATHN) and benzophenone 3 (BP3) in cooked and bioaccessibility fraction of European seabass burgers.

**Contaminant**	**Cooked European seabass**					
	**Linearity range ng/g (r)**	**Spiked level (ng/g)**	**Recovery (%) (Inter-day. %RSD)**	**Intra-day (%RSD)**	**LOD (ng/g)**	**LOQ (ng/g)**
**AHTN**	25-500 (0.9997)	25	78 (8)	10	0.6	2.5
		100	71 (6)	6		
		250	84 (7)	6		
**BP3**	150-5000 (0.9925)	150	77 (6)	8	6	20
		500	79 (8)	9		
		1000	81 (6)	8		
**Contaminant**	**Bioccessbility fraction**					
	**Linearity range ng/g (r)**	**Spiked level (ng/g)**	**Recovery (%) (Inter-day. %RSD)**	**Intra-day (%RSD)**	**LOD (ng/g)**	**LOQ (ng/g)**
**AHTN**	12.5-300 (0.9989)	15	87(15)	12	0.75	1.5
		50	71 (10)	5		
		100	80 (9)	6		
**BP3**	12.5-300 (0.9973)	15	78 (12)	11	2	5
		50	89 (13)	7		
		100	83 (11)	9		
